# Assessing the contribution of orthodontic profiles in predicting facial soft tissue thickness for forensic facial approximation

**DOI:** 10.1007/s00414-025-03542-x

**Published:** 2025-06-18

**Authors:** Muhammad Garry Syahrizal Hanafi, Hajime Utsuno, Shuuji Namiki, Nanami Aoki, Hisako Saitoh, Saki Minegishi, Sayaka Yamada, Yohsuke Makino, Hirotaro Iwase, Koichi Sakurada

**Affiliations:** 1 Department of Forensic Dentistry, Graduate School of Medical and Dental Sciences, Institute of Science Tokyo, 1-5-45 Yushima, Bunkyo-ku, Tokyo, 113-8510 Japan; 2 Department of Forensic Medicine, Graduate School of Medicine, The University of Tokyo, 7-3-1 Hongo, Bunkyo-ku, Tokyo, 113-0033 Japan; 3 Department of Legal Medicine, Graduate School of Medicine, Chiba University, 1-8-1 Inohana, Chuo-ku, Chiba, 260-8670 Japan

**Keywords:** Cephalic index, Facial soft tissue thickness, Japanese, Northwestern analysis, Skeletal class, Tweed analysis

## Abstract

**Background:**

Facial soft tissue thickness (FSTT) is essential for forensic facial approximation. Although its correlations with age, sex, and body mass index (BMI) are well documented, the potential correlations between FSTT and various orthodontic profiles—such as cephalic index (CI), skeletal class (SC), Tweed and Northwestern analyses—remain unexplored collectively. This study examined these correlations and their impact on FSTT prediction accuracy.

**Methods:**

We analyzed 103 postmortem computed tomography datasets from Japanese cadavers aged 18–86 years. Moderate-to-high multicollinearity was identified among orthodontic profile variables (SC, Tweed, and Northwestern) and addressed using principal component analysis (PCA), yielding two principal components (PC1 and PC2). Predictive formulas were constructed incorporating age, sex, BMI, CI, PC1, and PC2. To evaluate model performance, we conducted two comparative approaches: (1) comparing root mean squared error (RMSE) and mean absolute error (MAE) from the PCA-based regression model with those derived from holdout dataset’s BMI-based mean estimates, and (2) with primary dataset’s baseline regression model including only age, sex, and BMI, across all landmarks.

**Results and discussion:**

PCA reduced multicollinearity, retaining 77% of total data variability. Based on the two comparative approaches, the PCA-based regression model demonstrated marginal improvements in predictive accuracy, as indicated by slightly lower RMSE and MAE across most landmarks. It indicates a limited yet consistent benefit of using orthodontic profiles for enhancing model accuracy beyond basic demographic predictors.

**Conclusion:**

The inclusion of orthodontic profiles demonstrated modest improvements in predictive accuracy and may enhance the interpretive value of FSTT predictive models in forensic contexts.

**Clinical trial number:**

Not applicable.

**Supplementary Information:**

The online version contains supplementary material available at 10.1007/s00414-025-03542-x.

## Introduction

Forensic identification of unknown deceased individuals involves two main methods: primary and secondary. Primary identification is the principal approach based on scientific evidence encompassing DNA analysis, fingerprint analysis, and forensic odontology, by analyzing the odontogram. In contrast, secondary identification serves as a supporting method to complement the primary findings. It includes an examination of physical characteristics (such as height, weight, age estimation, birthmarks, scars, and tattoos) and personal items worn or found near the deceased (e.g., jewelry, clothing, and identification cards) [1, 2].

Meanwhile, facial reconstruction serves as a significant investigative method in forensic identification. Facial reconstruction is a method employed to estimate the facial characteristics of unidentified cranial remains. This technique, while not yielding a conclusive identification, can provide leads by visually indicating the potential facial appearances of the deceased. This technique is useful, particularly when traditional methods like fingerprints, dental records, or DNA analysis are unavailable or inconclusive. In the overall forensic identification process, facial reconstruction assists in the initial phases of forensic investigation by connecting the facial appearance with visual recognition [3, 4].

Facial reconstruction is performed by combining knowledge of the position and shape of the soft tissue structures in the face (eyes, nose, lips, and ears) with facial soft tissue thickness (FSTT), which serves as the foundation overlying the skeletal framework. Both aspects can be quantified and defined numerically to ensure the reliability and repeatability of facial reconstruction methods [5]. For forensic identification purposes, the FSTT is crucial for facial approximation and skull-photo superimposition. Specifically, FSTT provides insights into soft tissue depth at designated landmarks, enabling the accurate placement of reconstructed facial features in facial approximation. In skull-photo superimposition, FSTT assists in aligning skull images based on individual characteristics. Therefore, accurately predicting the FSTT during the facial approximation process is a critical step that can significantly affect the overall outcome [6].

Facial soft tissue thickness (FSTT) has been widely recognized to be influenced by body mass index (BMI), sex, and age. Among these three variables, BMI is considered the most strongly associated with FSTT, as increased BMI leads to greater fat deposition in the compartments among facial muscles, thereby increasing soft tissue thickness in those regions [7]. From a sex-based perspective, males are generally assumed to have higher FSTT than females, likely due to greater physical activity resulting in more developed musculature [7–9]. However, interestingly, a study by Stephan et al. in 2015 [10] suggested that after normalizing for BMI, females exhibit greater FSTT than males due to their generally lower body weight. Regarding age, FSTT tends to decrease over time as a consequence of reduced muscle tone and soft tissue deformation [7, 9]. Age-related changes in the FSTT are typically associated with variations in the volume and positioning of fat compartments, skin deformation leading to wrinkles, and reduced muscle tone [11, 12]. These three factors are further linked to genetic variation across populations [12, 13].

In addition to these factors, craniometric parameters have also been assumed to have a potential correlation with FSTT. Although several previous studies have demonstrated that one of the craniometric measurements—skeletal class (SC)—can enhance FSTT prediction [14–16], a study by Hona and Stephan in 2024 reported that the correlation between craniometric dimensions—specifically the linear chordal diameter of the skull—and FSTT is weak [17].

Despite these insights, many prior studies suffer from methodological constraints, including small sample sizes and non-random sampling, which may limit the generalizability and reliability of the regression outcomes [18]. By contrast, the present study utilized a relatively large sample of over 100 individuals, thereby enhancing statistical power and enabling more robust estimation of FSTT predictors.

In parallel, orthodontic profile assessments also incorporate cephalic index (CI) and lateral assessments such as Tweed and Northwestern methods, which have not been directly linked to the FSTT. The CI is a numerical value used to classify the shape of the head based on its width and length. Tweed analysis evaluates a theoretical diagnostic triangle formed by the frankfort horizontal plane (FHP), mandibular plane, and mandibular incisor plane, allowing practitioners to assess the severity and complexity of various malocclusion cases [19]. The Northwestern analysis involved a thorough examination of multiple angles in the maxillofacial region, including the NAP, SNA, SNB, SN to Go-Gn plane, FH to NP plane, and S-N-Gn angle. These angular assessments provide insight into the patient’s dentofacial profile and inform treatment planning [20, 21].

Although CI, Tweed, and Northwestern analyses offer a comprehensive method for evaluating craniofacial structures and serve as a potential predictor of FSTT, no study has explored the relationship between these orthodontic profiles and FSTT. Understanding this association may enhance the personalization of FSTT predictions. Moreover, integrating these profiles with SC is expected to improve FSTT prediction accuracy, particularly when dealing with complete skull remains.

Thus, this study aimed to evaluate the correlation between FSTT and orthodontic profiles. Subsequently, we determined whether the incorporation of these orthodontic profiles enhanced the accuracy of the FSTT prediction model.

## Materials and methods

### Study design and sample population

This study utilized a cross-sectional design to investigate the correlation between FSTT and orthodontic profile assessments (SC, CI, Tweed, and Northwestern profiles). We analyzed postmortem computed tomography (PMCT) data from 103 Japanese cadavers (62 males and 41 females) aged 18–86 years. Prior to autopsy, data were collected from the Department of Forensic Medicine at the University of Tokyo and the Department of Legal Medicine at Chiba University.

Only samples with the jaw and mouth in a closed position (centric occlusion) were examined because this is crucial for accurate orthodontic measurements. The exclusion criteria included cadavers in the decomposition phase, those with facial deformities or congenital malformations, presence or history of maxillofacial bone fractures, previous orthodontic treatments, and those with extensive metal restorations causing artifacts in radiographic images.

### Compliance with ethical standards

This study was conducted in accordance with the Declaration of Helsinki and was approved by the Ethics Committee of the Institute of Science Tokyo (formerly Tokyo Medical and Dental University) under approval number D2018-058-06 and The University of Tokyo under approval number 12,063-(2).

This study involved anonymized X-ray images of deceased individuals, ensuring no possibility of identification and eliminating the need for consent to participate or publish.

### Variables

The dependent variables in this study consisted of 36 FSTT landmarks, including 10 midline and 26 bilateral landmarks (Supplementary File 1). These landmarks align with those used in previous studies on the Japanese population [22].

There were 21 independent variables including age, sex, BMI, and orthodontic profile assessments (SC, CI, Tweed, and Northwestern) analyzed in this study (Supplementary File 1). Age was confirmed using medical records, personal data, or analyses based on anthropological assessments. Sex was determined through visual examination and cross-verified using medical records. BMI was calculated based on the length and weight of the cadavers. Meanwhile, the Tweed profile included three sub-variables, whereas the Northwestern profile comprised 13 subvariables.

### Data collection

#### PMCT protocol

At The University of Tokyo, a 16-row detector CT system (Eclos; Fujifilm Corporation, Tokyo, Japan) was used, with the scanning protocol set to a tube voltage of 120 kV, tube current of 200 mA, and slice thickness of 0.625 mm.

Chiba University employed a 64-row detector CT system (Supria Grande; Fujifilm Corporation, Tokyo, Japan) with a CT protocol featuring a tube voltage of 120 kV, tube current of 250 mA, and a slice thickness of 0.625 mm.

All facial soft tissue data taken in this study were obtained from cadavers in the supine position, which is common in medical imaging, especially for PMCT protocols. The potential influence of body position on FSTT measurements is acknowledged [23–25].

#### Measurements

PMCT imaging results were obtained as digital imaging and communications in medicine (DICOM) data consisting of hundreds or thousands of image slices for each sample. These DICOM files were reconstructed into a three-dimensional (3D) computed tomography (CT)/multiplanar image using the Pixmeo OsiriX MD software (version 13.0.3).

**Fig. 1:**
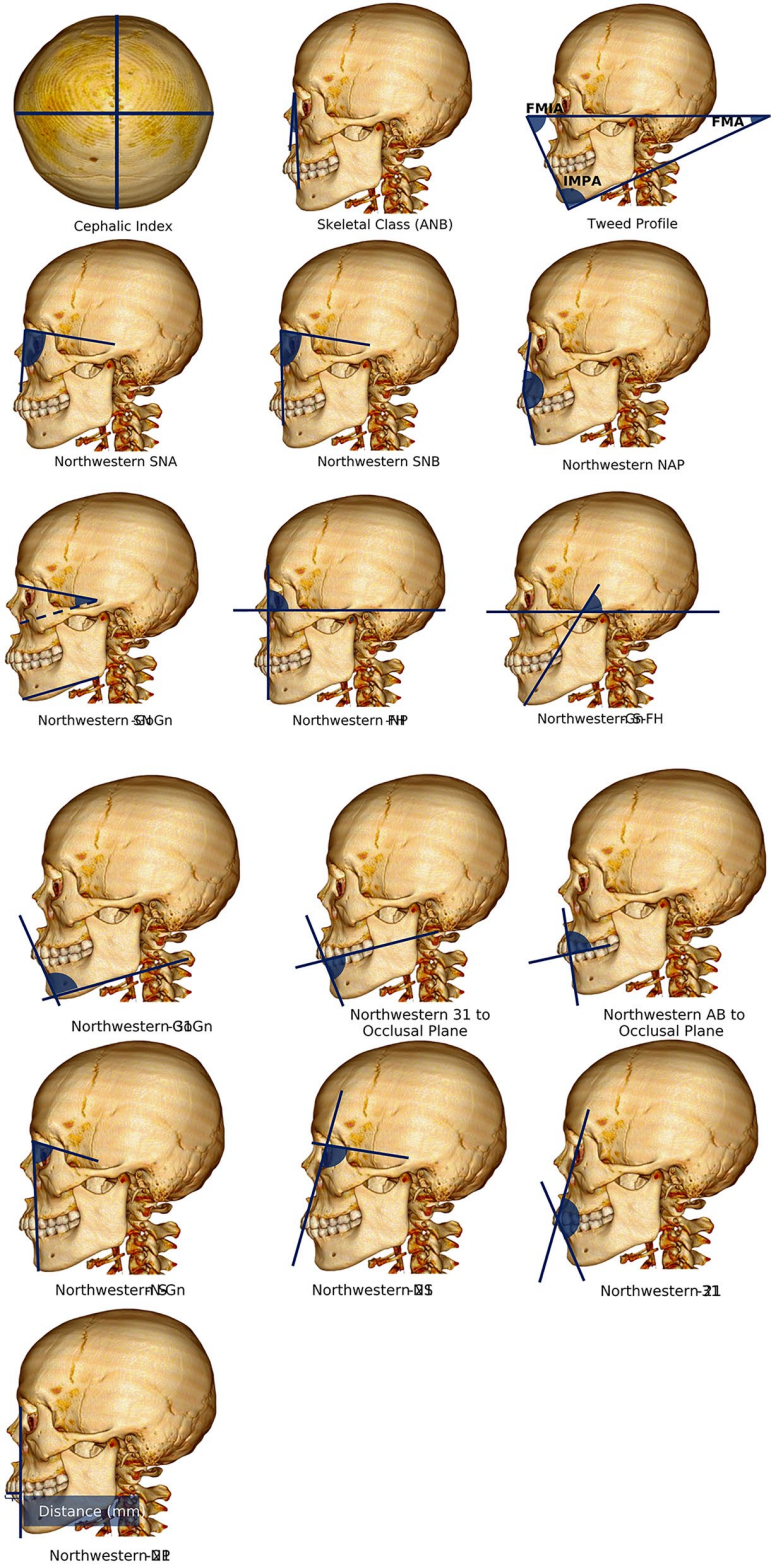
Orthodontic profile assessments measured

**Fig. 2:**
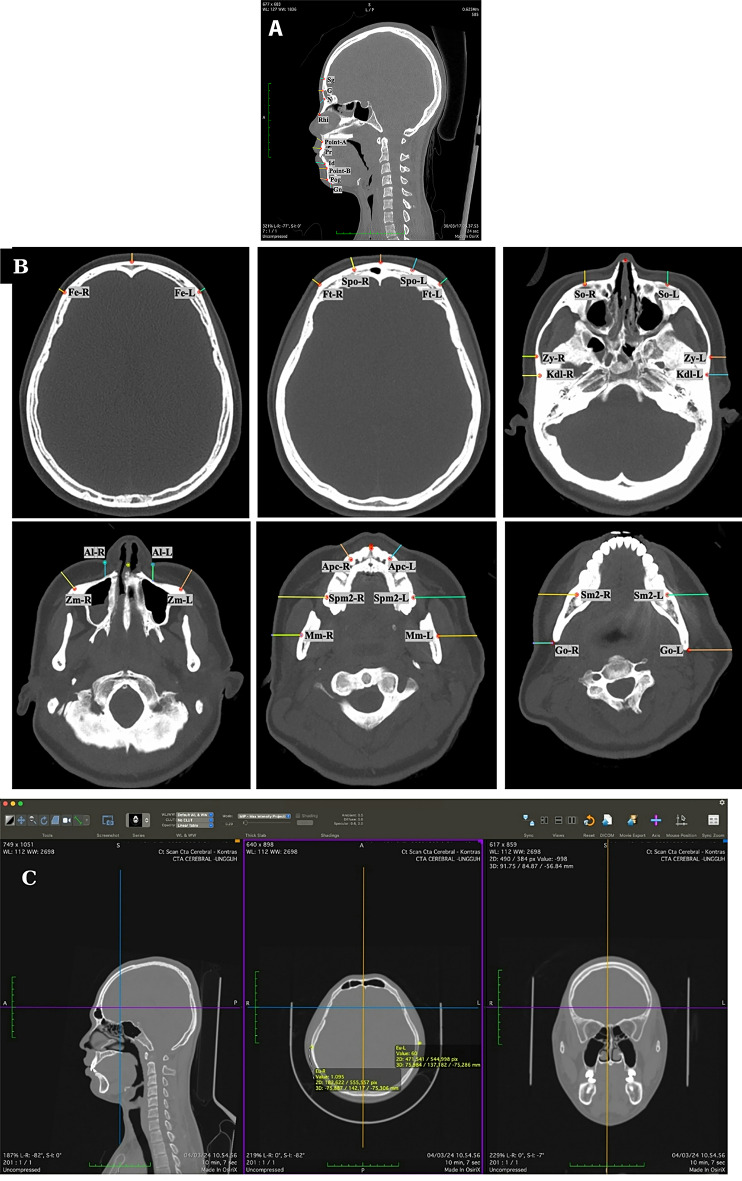
Landmarks measured and their direction. **a**) Midline landmarks; **b**) Bilateral landmarks; **c**) Multislice reconstruction

The orthodontic profile assessments and FSTT measurements at each landmark followed these steps. First, the 3D CT multiplanar image was aligned to the FHP by identifying the right and left porion points and the left suborbital. Second, each landmark was plotted to determine its specific location. Third, we measured the angle for each orthodontic profile assessment, as shown in Fig. 1. Finally, measurements were taken from each landmark to the outer edge of the soft tissue using the distance measurement tool in the OsiriX MD software to measure the FSTT. Figure 2 illustrates the distribution of the landmarks and the projection of the measurement directions of the FSTT for each landmark. For these Figures (i.e., Fig. 2a and b), a single multiplanar reconstruction screenshot was captured to display all relevant anatomical landmarks used in this study and how they were measured. Although the image may resemble raw orthoslices, it represents a synchronized view of the same reference point across three orthogonal planes (axial, coronal, and sagittal).

### Statistical analysis

A descriptive analysis was conducted to summarize the dataset. To assess the inter-observer measurement reliability, we performed the technical error of measurement (TEM). TEM was chosen because this method offers a direct estimate of the magnitude of measurement error and is considered more suitable than any other reliability tests, such as ICC, P values, and r [26]. Measurements were independently performed by two trained observers under the same conditions and following identical protocols. TEM was calculated for each variable using the standard formula stated by Fancourt and Stephan in 2018 [26]. Lower TEM values indicate higher measurement consistency between observers. In this study, TEM values in the range of 2 to 3 mm were interpreted as indicative of acceptable inter-observer reliability, based on conventional thresholds used in anthropometric and forensic morphometric research [26–28].

Data normality was tested using the Shapiro–Wilk test, and Pearson correlation analysis was applied considering the normal distribution. Correlation analyses evaluated the relationships between independent and dependent variables and tested the independence among the independent variables for multiple regression suitability. Continuous values were used for the CI and SC instead of categorical classifications to better analyze their relationships with FSTT.

Subsequently, based on the results of the correlation analysis among independent variables, we conducted a multicollinearity assessment by evaluating the variance inflation factor (VIF) using the following classification: “ideal” (< 5), “moderate” (5–10), and “high” (> 10) [29]. The VIF analyses revealed moderate to high values for SC, Tweed, and Northwestern variables. Principal component analysis (PCA) was applied to address multicollinearity and reduce dimensionality while retaining key information. From the PCA analysis, two principal components (PCs) were extracted, i.e., PC1 and PC2.

Using these two PCs, we first constructed a multiple regression model with the following independent variables: age, sex, BMI, CI, PC1, and PC2. We then calculated the root mean squared error (RMSE) and mean absolute error (MAE) to evaluate the accuracy of this model at each facial landmark. To further assess the impact of including orthodontic profiles on FSTT prediction, we compared the RMSE and MAE values from this model with those obtained from two alternative approaches:

1. Holdout dataset’s BMI-based mean estimates: To evaluate the generalizability and practical relevance of this comparison, an independent holdout dataset was utilized. This dataset consisted of PMCT scans from 142 cadavers with open-mouth positioning, which precluded orthodontic profiles analysis and therefore could not be used to construct or validate the regression model. However, it was suitable for calculating BMI-based mean FSTT values, which were then applied as predicted values for each corresponding group. These values were compared to the actual FSTT measurements to calculate RMSE and MAE. In this approach, we categorized the holdout samples into three BMI groups: underweight (< 18.5 kg/m^2^), normal weight (18.5–24.9 kg/m^2^), and overweight (> 25 kg/m^2^).
2. Primary dataset’s Baseline regression model: We developed an additional regression model using only three widely recognized predictors of FSTT—sex, age, and BMI—referred to as baseline variables [7, 8, 30] from the primary dataset. From this model, predicted FSTT values were generated for each landmark, followed by the calculation of RMSE and MAE. In addition to RMSE and MAE, we also conducted an R^2^ analysis to evaluate the proportion of variance in FSTT explained by both the PCA-based regression and the baseline regression model.

All statistical analyses were performed using Microsoft Excel (version 16.87) and SPSS (version 27.0), and a significance threshold of *p* < 0.05 was applied across all analyses conducted in this study to determine statistical significance.

## Results

### Descriptive statistics

Table 1 presents the descriptive analysis of the 103 cadavers in this study, comprising 62 males (mean age 47.3 ± 16.8 years) and 41 females (mean age 40.1 ± 18.8 years). The overall age range was 18–86 years, with an average age of 44.5 ± 17.9 years and a median age of 41 years. In terms of BMI, 47.6% were of normal weight (18.5–24.9 kg/m^2^, consisting of 26 males and 23 females), 17.5% were underweight (< 18.5 kg/m^2^, consisting of 8 males and 10 females), and 34.9% were overweight (> 25 kg/m^2^, consisting of 28 males and 8 females) (Supplementary File 2).

**Table 1:** Descriptive statistics

Variables	Mean	S.E. Mean*	Median	S.D.**	Min	Max
Age (years)	44.46	1.76	41	17.85	18	86
BMI (kg/m^2^)	22.76	0.47	21.97	4.82	11.75	38.92
Cephalic index (%)	84.06	0.48	84.10	4.91	69.64	96.24
Skeletal class/ANB (°)	4.97	0.26	4.90	2.65	-1.75	11.17

*S.E. Mean = Standard Error of the Mean

**S.D. = Standard Deviation

### Inter-observer analysis results

**Fig. 3:**
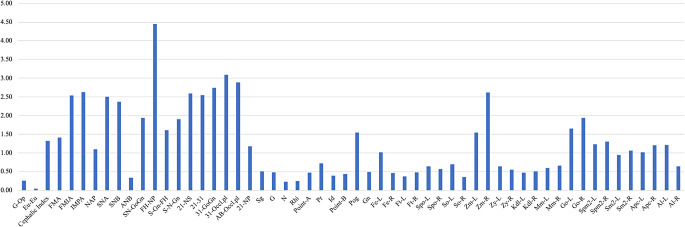
TEM results of the measured variables

TEM analysis was performed to assess the inter-observer reliability across 36 dependent and 21 independent variables. Approximately 90% of the variables exhibited TEM values below or around 2 to 2.5 mm, indicating high measurement consistency [26–28]. Only two variables, FH-NP (4.45) and 31-Occlusal Plane (3.09), prominently exceeded the threshold (Fig. 3).

### Correlation analysis between independent and dependent variables

**Fig. 4:**
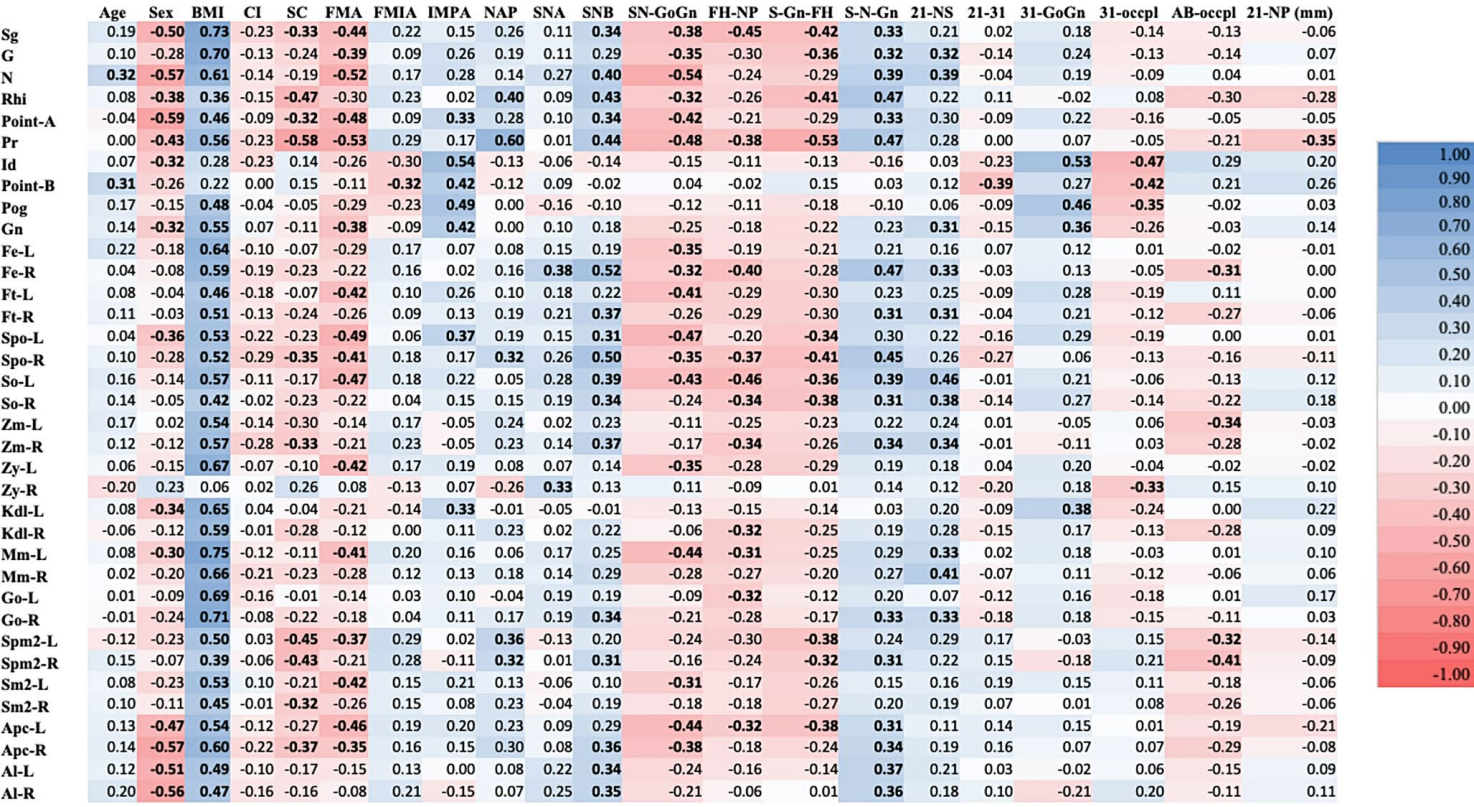
Visualization of correlation analysis between the independent variables and FSTT using a heat map. Bold numbers indicate that the correlation is significant (*p* < 0.05)

Figure 4 presents the results of the Pearson correlation analysis between the independent and dependent variables. The analysis revealed that 11 independent variables were positively correlated with FSTT across nearly all landmarks: age, BMI, FMIA, IMPA, SNA, SNB, S-N-Gn, 21-NS, 31-GoGn, and 21-NP. Conversely, 10 variables were negatively correlated with the FSTT: sex, CI, SC, FMA, SN-GoGn, FH-NP, S-Gn-FH, 21–31, 31-occlusal plane, and AB-occlusal plane.

BMI exhibited the strongest correlation with FSTT compared to the other independent variables. Among the 36 landmarks evaluated in this study, 33 showed a strong relationship with BMI. The next variable that correlated closely with FSTT across nearly all landmarks was sex, with males displaying a higher mean FSTT than females, especially at midline landmarks, the upper third of the face, and the subnasal area.

For the orthodontic profiles, SNB, SN-GoGn, and S-N-Gn emerged as the three variables most strongly correlated with FSTT. All three variables showed significant correlations primarily in the upper third of the face, midface, and cheek regions.

### Correlation analysis among independent variables

**Fig. 5:**
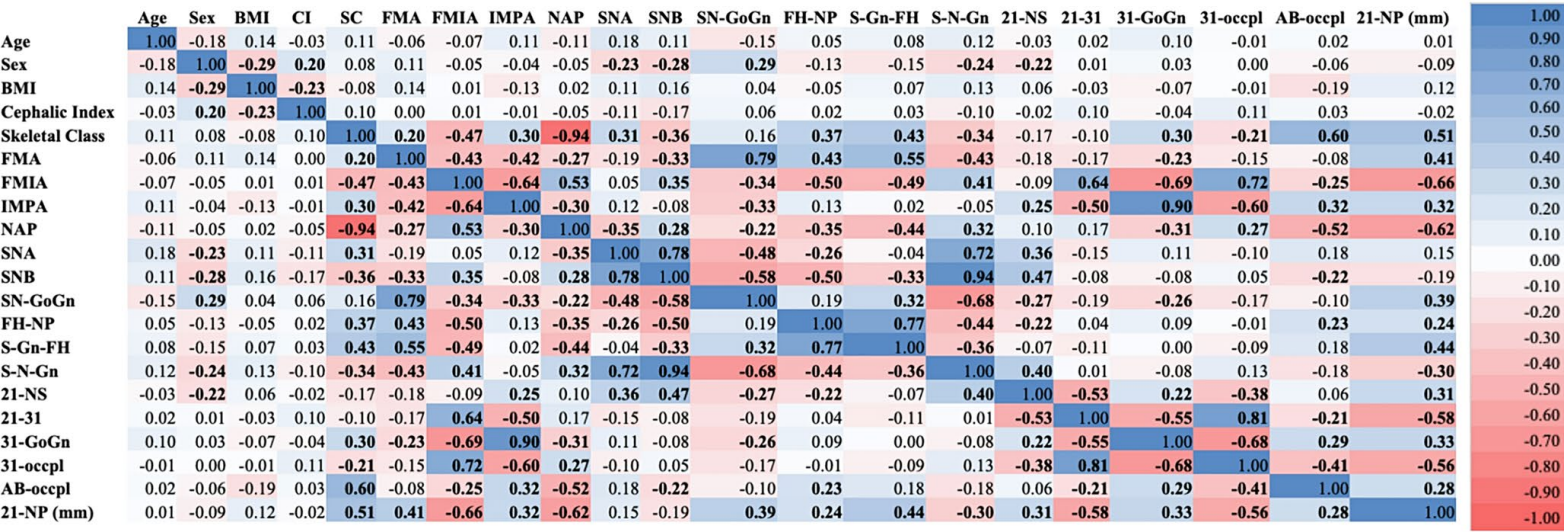
Visualization of correlation analysis among the independent variables using a heat map. Bold numbers indicate that the correlation is significant (*p* < 0.05)

Figure 5 shows the correlations among the independent variables. The orthodontic profile variables, particularly SC, Tweed, and Northwestern, exhibited strong intercorrelations, either positive or negative correlations. The highest positive correlation was observed between SNB and S-N-Gn (*r* = 0.94), while the highest negative correlation occurred between SC and NAP (*r* = -0.94). Additionally, FMIA and 21-NS had the highest number of significant correlations (*p* < 0.05) with the other variables, with a total of 14 significant intercorrelations.

### Multicollinearity analysis and principal component analysis

Considering the high correlations among independent variables, we conducted VIF analyses to identify the effect of multicollinearity within each variable. The results showed high VIF values for the orthodontic profile variables, particularly for SC, Tweed, and Northwestern (Supplementary File 1).

To address high multicollinearity, we performed PCA on those variables with high VIF values. Through 10 iterations of dimension reduction, eliminating variables with a measure of sampling adequacy (MSA) around 0.5 in each anti-image matrix, we obtained two PCs (PC1 and PC2) with a Kaiser-Meyer-Olkin (KMO) value of 0.79 and a total eigenvalue/variance of 76.6%. The KMO value indicates strong intervariable correlations, supporting the reliability of PCA and confirming that multicollinearity is no longer a significant issue. Meanwhile, the eigenvalue was ideal, while further variable reduction was avoided for two reasons: no MSA values in the anti-image table were below 0.5, and further reduction decreased the total variance to below 70%, compromising data representativeness [29, 31, 32].

These two PCs included the variables SC, FMIA, IMPA, 21–31, 31-GoGn, 31-Occlusal Plane, and 21-NP (Supplementary File 1). The first PC was influenced by Tweed and Northwestern variables, while the second was driven by SC and Northwestern variables. PC1 was predominantly influenced by broader variations, such as FMIA, IMPA, 21–31, 31-GoGn, and the 31-occlusal plane, while PC2 captured narrower variables driven by SC, FMIA, and 21-NP. This suggests that PC1 reflects patterns specific to the Tweed and Northwestern profiles, whereas PC2 captures patterns specific to the SC and Northwestern profiles.

### Multiple linear regression analysis for PCA model

PC1 and PC2 were used as new independent variables in the multiple linear regression analysis. Therefore, the predictive formula we developed consisted of baseline variables (age, sex, and BMI), CI, and the two PCs. We also conducted RMSE, MAE, and R^2^ metric analyses to evaluate the performance of the model (Table 2). Lower values of RMSE and MAE indicate better model performance, whereas higher R^2^ values reflect a greater proportion of variance explained by the model. From the metric analysis results, the Rhi landmark showed the lowest RMSE and MAE values, at 0.83 and 0.59, respectively. Meanwhile, the highest R² value was achieved at the Mm-L landmark, with a value of 0.61.

**Table 2:** Regression analysis of PCA models (age, sex, BMI, CI, PC1, and PC2)

Landmark	B0^a^	B1^b^	*P*	B2^c^	*P*	B3^d^	*P*	B4^e^	*P*	B5^f^	*P*	B6^g^	*P*	RMSE	MAE	*R* ^2^	*n*
Sg	2.608	0.000		-0.462		0.164	**	-0.007		-0.042		-0.073		1.09	0.81	0.39	100
G	1.862	-0.003		-0.385		0.149	**	0.003		-0.128		-0.031		0.99	0.74	0.38	100
N	7.573	0.000		-1.439	**	0.116	**	-0.024		-0.322	*	-0.129		1.41	1.05	0.38	100
Rhi	2.729	0.012	*	-0.376	*	0.048	*	-0.017		0.007		-0.178	*	0.83	0.59	0.25	100
Point-A	16.357	-0.046	**	-1.560	**	0.082	*	-0.043		-0.352	*	-0.594	**	1.63	1.32	0.42	98
Pr	14.836	-0.044	**	-1.259	**	0.160	**	-0.049		-0.125		-0.599	**	1.42	1.12	0.51	87
Id	12.975	-0.018		-1.491	**	0.135	*	-0.012		-0.310		0.093		2.09	1.72	0.22	94
Point-B	10.709	0.002		-0.668		0.131	**	-0.011		-0.266		0.300		1.74	1.37	0.21	98
Pog	4.345	-0.003		-0.487		0.295	**	0.012		-0.245		-0.081		2.60	1.99	0.24	100
Gn	-14.973	-0.004		-1.294	*	0.363	**	0.196	**	-0.497		-0.258		2.81	2.19	0.33	100
Fe-L	-1.167	0.000		-0.120		0.262	**	0.003		0.087		0.107		1.40	1.03	0.44	100
Fe-R	-0.161	-0.009		0.095		0.265	**	-0.008		0.013		0.019		1.34	1.00	0.45	100
Ft-L	2.673	-0.004		0.185		0.266	**	-0.031		0.011		0.103		1.57	1.16	0.39	100
Ft-R	1.817	-0.011		0.187		0.273	**	-0.018		-0.015		-0.009		1.62	1.20	0.38	100
Spo-L	5.604	-0.004		-0.702	*	0.209	**	-0.019		-0.195		-0.153		1.53	1.27	0.37	100
Spo-R	6.577	-0.009		-0.602		0.213	**	-0.028		-0.213		-0.130		1.46	1.23	0.39	100
So-L	2.159	0.009		0.549		0.325	**	-0.037		-0.097		-0.025		1.77	1.39	0.43	100
So-R	2.021	0.007		0.446		0.281	**	-0.020		-0.138		-0.087		1.92	1.50	0.32	100
Zm-L	0.333	-0.041	*	0.703		0.605	**	0.033		0.407		-0.110		2.60	1.98	0.53	100
Zm-R	8.634	-0.036		0.157		0.529	**	-0.041		0.172		-0.297		3.05	2.33	0.41	100
Zy-L	-12.631	-0.027		1.570	*	0.618	**	0.074		-0.147		0.033		2.72	1.94	0.50	99
Zy-R	-8.006	-0.031		2.264	**	0.573	**	0.033		0.046		-0.159		2.79	2.07	0.46	100
Kdl-L	-15.222	-0.041		-0.358		0.910	**	0.153		-0.547		0.693		3.70	2.85	0.56	99
Kdl-R	-8.411	-0.056	*	0.259		0.857	**	0.094		-0.471		0.162		4.02	3.15	0.48	100
Mm-L	-8.812	-0.013		0.779		1.078	**	0.085		-0.320		0.018		3.86	2.99	0.61	99
Mm-R	4.653	-0.047		0.020		0.869	**	0.017		-0.483		-0.488		4.11	3.20	0.49	100
Go-L	-14.835	-0.011		2.521		1.319	**	-0.014		-0.463		0.798		5.48	4.33	0.50	98
Go-R	-18.211	-0.022		0.287		1.141	**	0.132		-0.618		-0.588		5.72	4.58	0.42	99
Spm2-L	-5.496	-0.018		-0.081		1.043	**	0.195		0.625		-1.502	*	4.87	3.84	0.51	70
Spm2-R	1.014	-0.023		1.920		0.942	**	0.113		0.743		-1.588	*	6.07	4.82	0.33	70
Sm2-L	-15.293	0.013		-0.733		0.937	**	0.318	*	-0.442		-0.873		4.95	3.90	0.43	65
Sm2-R	-0.043	0.035		0.589		0.824	**	0.146		-0.563		-1.151		5.20	4.09	0.33	64
Apc-L	12.336	-0.029	*	-1.411	**	0.230	**	-0.025		-0.093		-0.341		2.03	1.65	0.36	97
Apc-R	12.660	-0.027	*	-2.060	**	0.210	**	-0.016		-0.152		-0.548	*	2.11	1.60	0.40	95
Al-L	4.950	-0.028		-0.811		0.343	**	0.045		-0.307		-0.388		3.27	2.81	0.22	100
Al-R	6.541	-0.024		-1.358		0.318	**	0.034		0.014		-0.270		3.16	2.61	0.24	100

^a^constant; ^b^age; ^c^sex; ^d^BMI; ^e^cephalic index; ^f^PC1; ^g^PC2; *significant at the 0.05 level; **significant at the 0.01 level

The PC values (PC1 and PC2) were calculated by summing the product of the loadings for each independent variable with the actual value of that variable for a given subject. This calculation yielded the weighted contribution of each independent variable to the respective PC. Therefore, the formula for calculating PCs is expressed as follows:

PC1 =– (SC × 0.04) + (FMIA × 0.72)– (IMPA × 0.84) + (21–31 × 0.80)– (31-GoGn × 0.88) + (31-Occl. plane × 0.86)– (21-NP × 0.38) (1).

PC2 = (SC x 0.89)– (FMIA × 0.55) + (IMPA × 0.14)– (21–31 × 0.20) + (31-GoGn × 0.15)– (31-Occl. plane × 0.25) + (21-NP × 0.77) (2).

After obtaining the PC1 and PC2 values for each landmark, these values were incorporated into the regression formula, which is structured as follows:

FSTT = B0 + (age × B1) + (sex × B2) + (BMI × B3) + (CI × B4) + (PC1 × B5) + (PC2 × B6) (3).

### Multiple linear regression analysis for baseline model

We also developed the baseline model using three variables (age, sex, and BMI) for each landmark. These models also used RMSE, MAE, and R^2^ as evaluation metrics (Supplementary File 1). The results were similar to those of the PCA models, with the lowest RMSE and MAE values observed at the Rhi landmark (0.86 and 0.64, respectively), whereas the highest R^2^ value was recorded at the Mm-L landmark (0.61). BMI was correlated with all landmarks, sex with 10 landmarks, and age with 7 landmarks. The regression equation structure was similar to that of Eq. (3) but without CI, PC1, and PC2.

### Comparison of FSTT prediction accuracy: regression models vs. holdout samples BMI-based mean

A comparative analysis was conducted between the FSTT predictions derived from the primary dataset’s PCA-based regression model and those estimated using mean BMI values from the holdout dataset (Supplementary File 2). The RMSE and MAE values consistently showed a modest improvement in the PCA-based regression model predictions across the majority of landmarks. The most notable improvement in RMSE and MAE was observed at the Point-A landmark, showing a reduction of 2.55 mm and 1.56 mm, respectively.

However, middle third landmark (Apc-L/R), some lower third landmarks (Id, Point-B, Pog, and Gn), and four cheek landmarks (Spm2-L, Spm2-R, Sm2-L, and Sm2-R), demonstrated higher values of either RMSE, MAE, or both standard errors in the PCA-based regression model, indicating reduced predictive accuracy for these specific areas using the regression approach.

However, overall, these results suggest that PCA-based regression models are slightly more accurate in FSTT estimates compared to predictions based solely on BMI-based mean values.

### Comparison of FSTT prediction accuracy: between the two linear regression models

The comparison of RMSE, MAE, and R² showed marginal differences between the PCA-based and baseline regression models (Supplementary File 2). In the RMSE analysis, the PCA model had slightly smaller values across almost all landmarks except for Ft-L, Zm-R, and Mm-L, which had equal values, and Pog, Fe-R, Zy-L, and Zy-R, which had larger values. For MAE, the PCA model also generally showed smaller values, except for Ft-L (equal values) and Pog, Fe-R, Zm-R, Zy-L, Kdl-R, Mm-L, Go-L, and Smp2-L (larger values). In the R² analysis, the PCA model showed larger values for half of the landmarks. Meanwhile, others, such as Fe-L, Spo-R, Mm-L, and Go-L, had equal values, and Sg, G, Pog, Fe-R, Ft-L, Ft-R, So-L, So-R, Zm-R, Zy-L, Zy-R, Kdl-R, Mm-R, and Go-R had smaller values.

After adding the orthodontic profile variables to the regression formula, landmarks in the cheek area (Spm2 and Sm2 on both sides) showed the most significant improvements. In the RMSE analysis, Spm2-L, Spm2-R, Sm2-L, and Sm2-R exhibited reductions of 1.00, 0.22, 0.24, and 0.14, respectively. In the MAE analysis, the values that decreased for these landmarks were 0.72, 0.21, 0.29, and 0.06, respectively. For R², the observed increases were 0.21, 0.06, 0.07, and 0.03, respectively. We also observed a large metric difference in the upper lip region, especially at the Point-A and Pr landmarks. For Point-A, the RMSE change was 0.17, MAE was 0.14, and R^2^ was 0.12. For Pr, the metric changes were 0.19, 0.17, and 0.11 for RMSE, MAE, and R^2^, respectively. These values exceeded the average changes for each metric for all landmarks, where the change in mean RMSE was 0.09, MAE was 0.07, and R² was 0.02.

## Discussion

This study utilized PMCT data collected in the supine position, which has been shown to influence FSTT in areas such as the cheeks and nasolabial folds due to gravitational effects [23]. However, literature indicates that 82.4–86.7% of facial surface areas remain within an acceptable error range (± 2 mm), supporting the anatomical validity of most landmarks under this position [23–25]. Nevertheless, the application of adjusted values to minimize the limitations associated with the supine position, as suggested by Munn and Stephan [24], is recommended for future studies. Moreover, it would be beneficial to develop population-specific adjusted values for the Japanese population to ensure more accurate and appropriate application of FSTT estimates in this demographic [22].

The use of cadaveric samples for observing FSTT is often associated with postmortem soft tissue changes, such as shrinkage, dehydration, and loss of muscle tone due to the postmortem interval. However, these changes are typically observed in cadavers examined more than 24 h after death or in those not stored in mortuary refrigerators at 4^o^C, where tissue distortion may occur. In this study, the average postmortem CT scans were performed within 24 h after death. For cases scanned after this period, the cadavers had been properly stored in refrigerated mortuary conditions, thus minimizing or even eliminating soft tissue distortion. This has been supported by a study conducted by Tanaka et al. in 2020, which demonstrated no significant differences in FSTT between cadavers and living persons in the same population as that of the current study, Japanese [22].

Interestingly, although age is widely considered an important factor influencing FSTT, our analysis found no significant correlation at most landmarks. This contrasts with the findings in other populations, such as those in Belgium [8], Sri Lanka [11], China [33], and Pakistan [34]. However, our findings align with those of studies from Italy [7] and the same location as this study, Japan [20, 22], highlighting population-specific variations. In this study, the impact of age on the FSTT was surpassed by nearly all orthodontic profile measurements. This may suggest that the orthodontic profile information potentially has greater predictive value for FSTT than age across most landmarks.

We conducted a correlation analysis among the independent variables to identify potential multicollinearity. The results revealed significant intercorrelations, particularly among the SC, Tweed, and Northwestern profiles. In contrast, CI showed fewer intercorrelations with other variables, likely because it measures the horizontal and longitudinal dimensions of the head, whereas the three orthodontic profiles analyzed in this study primarily involve the lateral dimensions [18]. These findings indicate a high potential for multicollinearity among independent variables, as confirmed by VIF analysis, where nine variables had a VIF > 10 and six had a VIF > 5. Addressing multicollinearity is essential before performing multiple linear regression, because it can lead to unstable coefficients and reduced model predictive power [31]. We selected PCA to address this multicollinearity because it transforms correlated variables into a smaller set of uncorrelated components, preserving most of the original data’s variability. This approach enables the inclusion of orthodontic profile information in the regression model without compromising the model’s stability and interpretability [32, 35].

The findings from our comparative analysis provide a more nuanced understanding of the potential use of regression-based models in estimating FSTT. Predictions based on PCA regression in the primary dataset showed slightly lower RMSE and MAE values compared to the BMI-based mean estimates in the holdout dataset, indicating an improvement in predictive accuracy. BMI was selected as the reference predictor in the mean-based model due to its well-established association with FSTT, as confirmed by both our correlation analysis and previous studies [7, 8, 30]. However, although the PCA-based regression model showed generally better performance, the extent of improvement remained modest, ranging from 0.9 to 2.6 mm, and varied depending on anatomical region. Thus, the practical contribution of this improvement to facial approximation remains debatable.

Notably, improvements with RMSE and MAE reductions greater than 0.5 mm were more frequently observed at middle third landmarks such as Point-A, Pr, So-L/R, Zy-L/R, Kdl-L/R, and Apc-L/R, as well as bilateral landmarks like Mm-L/R and Go-L/R. The most significant improvement was seen at Point-A (2.55 mm), possibly indicating that the incorporation of orthodontic profile variation via PCA helped improve estimation accuracy at this particular site. This aligns with anatomical expectations, as Point-A is influenced by skeletal classification and lateral orthodontic profile differences. The other middle third landmarks’ improvements ranged between 0.5 and 1 mm, suggesting that PCA-based regression may help capture facial shape variation not fully reflected in BMI-based estimates. However, given the relatively small numerical gains, these findings should be interpreted conservatively and validated in future studies with larger and more diverse samples.

Contrary to initial expectations, several landmarks in the lower third and bilateral regions showed higher RMSE and/or MAE values in the PCA-based regression model than in the BMI-based mean model. For instance, the cheek regions (Spm2 and Sm2) exhibited discrepancies ranging from 0.8 to 2 mm in RMSE and 2–3 mm in MAE. One possible explanation is that the lower third area may have been affected by the subjects’ open-mouth posture in the holdout samples. As reported by Tanaka et al. in 2020 [22], while FSTT values between living individuals and cadavers were largely similar in the Japanese population, differences were noted in the lower midfacial area, likely due to differences in soft tissue tension under open versus closed mouth positions. For further research, it may be advisable to ensure closed-mouth samples when using BMI-based reference values to improve comparability. Furthermore, the relatively better performance of BMI-based estimates in the cheek areas suggests that in these regions, the mean BMI may serve as a more effective predictor, as the cheeks are among the facial areas with the highest fat distribution. Therefore, increases in BMI—which correspond to greater fat deposition—may more directly reflect increases in FSTT in these anatomical landmarks [36, 37]. These varied results suggest that regression-based approaches incorporating orthodontic profiles may offer more personalized and potentially more accurate FSTT estimations in certain regions.

Likewise, a comparison of the linear regression models between the PCA-based and baseline regression models indicated that incorporating orthodontic profiles through PCA also yielded marginally improved model performance in predicting FSTT across several landmarks. Although the PCA model yielded reduced RMSE and MAE values compared to the baseline model at most landmarks—with the cheek and upper lip areas becoming the most pronounced enhancements—the variations were typically minimal, which was frequently below 0.07 mm. In addition, RMSE and MAE of PCA-based regression in some bilateral landmarks, such as Fe-R, Ft-L, and Zm-R, showed equal or worse performance, while the contralateral sides showed better, indicating the effect of bilateral discrepancies due to the supine positioning of cadavers [24].

In conclusion, while the inclusion of orthodontic profiles led to minor reductions in RMSE and MAE, these improvements were limited in magnitude and may not be practically significant. Nevertheless, orthodontic profiles may contribute to a more nuanced understanding of FSTT variation and warrant further exploration as supplementary variables in predictive models for forensic applications.

### Limitation

One notable limitation of this study is the absence of a separate testing set to independently validate the regression models. This decision was primarily driven by practical constraints. Specifically, the collection of samples with a closed-mouth condition was particularly challenging. While open-mouth postures are common in cadaver-based datasets, closed-mouth samples suitable for orthodontic profiles measurement are limited. In our dataset, although we successfully collected 103 samples, several of these did not have all target landmarks observable, particularly in the cheek region (e.g., Spm2 and Sm2). This further reduced the number of complete and usable data points per landmark. Dividing an already limited dataset into separate training and testing subsets would have likely compromised the robustness of the regression models due to insufficient sample size, especially for more variable or less frequently observable landmarks. As such, we opted to maximize the use of all available data for model development to strengthen the reliability of the regression analysis, while acknowledging that this may limit the generalizability of the findings. Future studies with access to larger, high-quality datasets, ideally with consistent closed-mouth conditions and full landmark visibility, are recommended to enable more rigorous model validation using independent testing sets. However, as an alternative to an independent testing set, we employed BMI-based mean estimates as a reference model to evaluate the predictive capability of our regression model on a separate holdout dataset. This approach allowed us to assess the relative improvement in prediction accuracy without further subdividing the dataset, which would have reduced statistical power due to sample limitations.

Second, the improvement in RMSE and MAE values observed in this study was relatively small, indicating limited practical impact. Therefore, further confirmatory studies involving larger and more diverse populations are needed to validate the robustness and generalizability of the findings.

Third, despite the justification for using supine-based samples in this study, it still has the potential to influence FSTT due to gravitational effects. Previous studies have shown that FSTT shifts inferiorly and laterally under gravity when supine, with more pronounced changes observed in older individuals. Although most facial landmarks remain within a small range of error compared to upright position, significant displacement has been reported in up to 52% of bilateral and 12% of midsagittal landmarks. These positional effects may introduce measurement bias and should be acknowledged when applying the findings to forensic settings.

## Electronic supplementary material

Below is the link to the electronic supplementary material.

Supplementary Material 1

Supplementary Material 2

## Data Availability

The datasets generated and analyzed during the study are available from the corresponding author upon reasonable request. The supplementary data that is not included in the manuscript will be available online.

## Code Availability

Code availability: not applicable.

## References

[1] Roux C, Crispino F, Ribaux O (2012) From forensics to forensic science. Curr Iss Crim Justic 24:7–24

[2] Maras M-H, Miranda MD (2019) Forensic science. In: Marciano A, Ramello GB (eds) Encyclopedia of law and economics. Springer New York, New York, NY, pp 892–896

[3] Evison MP, Iwamura ESM, Guimarães MA, Schofield D (2016) Forensic facial reconstruction and its contribution to identification in missing person cases. Handbook of missing persons. Springer International Publishing, pp 427–441

[4] Stephan CN (2015) Facial Approximation-From facial reconstruction synonym to face prediction paradigm. J Forensic Sci 60:566–571 25703265 10.1111/1556-4029.12732

[5] Uma Maheswari T, Krishnan M (2019) Forensic facial reconstruction. Int J Forensic Odontol 4:1

[6] Moritsugui DS, Fugiwara FVG, Vassallo FNS, Mazzilli LEN, Beaini TL, Melani RFH (2022) Facial soft tissue thickness in forensic facial reconstruction: impact of regional differences in Brazil. PLoS ONE. 10.1371/journal.pone.0270980 35839226 10.1371/journal.pone.0270980 PMC9286276

[7] Piombino P, Esposito E, Committeri U et al (2023) Facial soft tissue thickness measurement method and relationship with BMI, age and sex. J Stomatol Oral Maxillofac Surg. 10.1016/j.jormas.2023.101420 36758899 10.1016/j.jormas.2023.101420

[8] de Greef S, Vandermeulen D, Claes P, Suetens P, Willems G (2009) The influence of sex, age and body mass index on facial soft tissue depths. Forensic Sci Med Pathol 5:60–65 19437147 10.1007/s12024-009-9085-9

[9] De Donno A, Mele F, Angrisani C, Maselli R, Cozzolino M, Pedote P, Introna F, Santoro V (2022) Facial approximation for identification purposes: soft tissue thickness in a Caucasian population. Sex and Age-Related variations. J Forensic Odontostomatol 40:34–41 35499535 PMC10228186

[10] Stephan CN, Preisler R, Bulut O, Bennett M (2016) Turning the tables of sex distinction in craniofacial identification: why females possess thicker facial soft tissues than males, not vice versa. Am J Phys Anthropol 161:283–295 27324815 10.1002/ajpa.23029

[11] Sandamini H, Jayawardena A, Batuwitage L, Rajapakse R, Karunaratna D, Vidanapathirana M, Pallewatte A (2018) Facial soft tissue thickness trends for selected age groups of Sri Lankan adult population. Forensic Sci Int 293:102.e1-102.e11 10.1016/j.forsciint.2018.10.001 30391103

[12] Rohrich RJ, Pessa JE (2007) The fat compartments of the face: anatomy and clinical implications for cosmetic surgery. Plast Reconstr Surg 119:2219–2227 17519724 10.1097/01.prs.0000265403.66886.54

[13] Baillie LJ, Mirijali SA, Niven BE, Blyth P, Dias GJ (2015) Ancestry and BMI influences on facial soft tissue depths for A cohort of Chinese and caucasoid women in dunedin, new Zealand. J Forensic Sci 60:1146–1154 26260028 10.1111/1556-4029.12799

[14] Park E, Chang J, Park J (2023) Facial soft tissue thickness differences among three skeletal classes in Korean population using CBCT. Int J Environ Res Public Health. 10.3390/ijerph20032658 36768023 10.3390/ijerph20032658 PMC9914978

[15] Sarilita E, Rynn C, Mossey PA, Black S, Oscandar F (2020) Facial average soft tissue depth variation based on skeletal classes in Indonesian adult population: A retrospective lateral cephalometric study. Leg Med. 10.1016/j.legalmed.2019.101665 10.1016/j.legalmed.2019.101665 31945677

[16] Utsuno H, Kageyama T, Uchida K, Kibayashi K (2014) Facial soft tissue thickness differences among three skeletal classes in Japanese population. Forensic Sci Int 236:175–180 24509238 10.1016/j.forsciint.2013.12.040

[17] Hona TWPT, Stephan CN (2025) Correlations of facial soft tissue thicknesses with craniometric dimensions improve craniofacial identification estimates: fact or fiction? J Forensic Sci. 10.1111/1556-4029.15694 39740019 10.1111/1556-4029.15694 PMC11874240

[18] Stephan CN, Sievwright E (2018) Facial soft tissue thickness (FSTT) Estimation models—And the strength of correlations between craniometric dimensions and FSTTs. Forensic Sci Int 286:128–140 29574348 10.1016/j.forsciint.2018.03.011

[19] Kumari L, Das A (2017) Determination of tweed’s cephalometric norms in Bengali population. Eur J Dent 11:305–310 28932138 10.4103/ejd.ejd_274_16 PMC5594957

[20] Yamagata S, Nishiura A, Hosoyama C, Nakayama Y, Yasui K, Morikuni H, Matsumoto N (2023) Cephalometric standards for Japanese adults with skeletal class I craniofacial morphology. J Osaka Dent Univ 1:179–186

[21] Zhu C, Hosoyama C, Nakayama Y, Yasui K, Morikuni H, Nishiura A, Matsumoto N (2022) Cephalometric analysis for Chinese adults with skeletal 1 craniofacial morphology. J Osaka Dent Univ 1:71–77

[22] Tanaka C, Utsuno H, Makino Y, Minegishi S, Ota J, Iwase H, Sakurada K (2020) Facial soft tissue thickness of the Japanese population determined using post mortem computed tomography images. Forensic Imaging. 10.1016/j.fri.2020.200423

[23] Ozsoy U, Sekerci R, Ogut E (2015) Effect of sitting, standing, and supine body positions on facial soft tissue: detailed 3D analysis. Int J Oral Maxillofac Surg 44:1309–1316 26116065 10.1016/j.ijom.2015.06.005

[24] Munn L, Stephan CN (2018) Changes in face topography from supine-to-upright position—And soft tissue correction values for craniofacial identification. Forensic Sci Int 289:40–50 29852367 10.1016/j.forsciint.2018.05.016

[25] Bulut O, Jessica Liu CY, Koca F, Wilkinson C (2017) Comparison of three-dimensional facial morphology between upright and supine positions employing three-dimensional scanner from live subjects. Leg Med 27:32–37 10.1016/j.legalmed.2017.06.002 28675828

[26] Fancourt HSM, Stephan CN (2018) Error measurement in craniometrics: the comparative performance of four popular assessment methods using 2000 simulated cranial length datasets (g-op). Forensic Sci Int 285:162–171 29501053 10.1016/j.forsciint.2018.02.008

[27] Mony PK, Swaminathan S, Gajendran JK, Vaz M (2016) Quality assurance for accuracy of anthropometric measurements in clinical and epidemiological studies [Errare humanum est = to err is human]. Indian J Community Med 41:98–102 27051083 10.4103/0970-0218.173499 PMC4799648

[28] Adão T, De Oliveira P, Perini TA, Lameira De Oliveira G, Dos J, Ornellas S Palha de Oliveira F technical error of measurement in anthropometry *

[29] Gwelo AS (2019) Principal components to overcome multicollinearity problem. Oradea J Bus Econ 4:79–91

[30] Nourmohammadi MJ, Ahmadi SAY, Rezaian J (2023) Structural equation modelling to estimate facial soft tissue thickness parameters based on ethnicity, gender and body mass index: a secondary study on an Iranian dataset. Surg Radiol Anat 45:739–746 37087723 10.1007/s00276-023-03147-2

[31] Perez LV (2017) Principal Component Analysis to Address Multicollinearity. Thesis, Whitman College

[32] Navelski J, Odongo K (2021) Making use of PCA in the presence of multicollinearity: an application to predicting body fat percentage. Department of Mathematics and Statistics & The School of Economic Sciences Washington State University. Accessed 12 January 2025

[33] Chen F, Chen Y, Yu Y, Qiang Y, Liu M, Fulton D, Chen T (2011) Age and sex related measurement of craniofacial soft tissue thickness and nasal profile in the Chinese population. Forensic Sci Int 212:272e1–272e6 10.1016/j.forsciint.2011.05.027 21715112

[34] Jeelani W, Fida M, Shaikh A (2017) Age and sex-related variations in facial soft tissue thickness in a sample of Pakistani children. Aust J Forensic Sci 49:45–58

[35] Shui W, Zhou M, Maddock S, He T, Wang X, Deng Q (2017) A PCA-Based method for determining craniofacial relationship and sexual dimorphism of facial shapes. Comput Biol Med 90:33–49 28918063 10.1016/j.compbiomed.2017.08.023

[36] Toneva D, Nikolova S, Georgiev I, Harizanov S, Zlatareva D, Hadjidekov V, Lazarov N (2018) Facial soft tissue thicknesses in Bulgarian adults: relation to sex, body mass index and bilateral asymmetry. Folia Morphologica (Poland) 77:570–582 10.5603/FM.a2017.0114 29235090

[37] Jeong SM, Lee DH, Rezende LFM, Giovannucci EL (2023) Different correlation of body mass index with body fatness and obesity-related biomarker according to age, sex and race-ethnicity. Sci Rep. 10.1038/s41598-023-30527-w 36859451 10.1038/s41598-023-30527-w PMC9977890

