# Supplementary material for: Assessing the contribution of orthodontic profiles in predicting facial soft tissue thickness for forensic facial approximation
Source: Int J Legal Med. 2025 Jun 18;139(6):2951–64. doi: 10.1007/s00414-025-03542-x (PMC12532767; doi:10.1007/s00414-025-03542-x)
Supplement: Supplementary file 1 — Supplementary Material 1 [file 414_2025_3542_MOESM1_ESM.docx]

**SUPPLEMENTARY FILE 1**

| Supplementary Table 1. Landmarks measured | | |
| --- | --- | --- |
| **No** | **Landmark** | **Abbreviation** |
| 1 | Supraglabella | Sg |
| 2 | Glabella | G |
| 3 | Nasion | N |
| 4 | Rhinion | Rhi |
| 5 | Point-A/Subspinale/Mid-philtrum | Point-A |
| 6 | Prosthion | Pr |
| 7 | Infradentale | Id |
| 8 | Point-B/Supramentale | Point-B |
| 9 | Pogonion | Pog |
| 10 | Gnathion | Gn |
| 11 | Left Frontal Eminence | Fe-L |
| 12 | Right Frontal Eminence | Fe-R |
| 13 | Left Fronto Temporale | Ft-L |
| 14 | Right Fronto Temporale | Ft-R |
| 15 | Left Supra Orbitale | Spo-L |
| 16 | Right Supra Orbitale | Spo-R |
| 17 | Left Sub Orbitale | So-L |
| 18 | Right Sub Orbitale | So-R |
| 19 | Left Zygomaxillare | Zm-L |
| 20 | Right Zygomaxillare | Zm-R |
| 21 | Left Zygion | Zy-L |
| 22 | Right Zygion | Zy-R |
| 23 | Left Condylion Laterale | Kdl-L |
| 24 | Right Condylion Laterale | Kdl-R |
| 25 | Left Mid Masseteric | Mm-L |
| 26 | Right Mid Masseteric | Mm-R |
| 27 | Left Gonion | Go-L |
| 28 | Right Gonion | Go-R |
| 29 | Left Supra Molar 2 | Spm2-L |
| 30 | Right Supra Molar 2 | Spm2-R |
| 31 | Left Sub Molar 2 | Sm2-L |
| 32 | Right Sub Molar 2 | Sm2-R |
| 33 | Left Apex of Canine | Apc-L |
| 34 | Right Apex of Canine | Apc-R |
| 35 | Left Alare | Al-L |
| 36 | Right Alare | Al-R |

| Supplementary Table 2. Orthodontic profile acronyms and definitions | |  |
| --- | --- | --- |
| **Profile** | **Acronyms** | **Definition** |
| **CI** | Cephalic Index | Ratio of head width to head length. [(Left Eurion-Right Eurion) / (Glabella-Opistochranion) x 100] |
| **SC** | Skeletal Class | Measured by ANB, the difference between the SNA and SNB angles |
| **Tweed** | | |
| **FMA** | Frankfort Mandibular Plane Angle | The angle between the mandibular plane and the Frankfort Horizontal Plane (FHP) |
| **FMIA** | Frankfort Mandibular Incisor Angle | The angle between the mandibular incisor and the FHP |
| **IMPA** | Incisor Mandibular Plane Angle | The angle between the lower incisor and the mandibular plane |
| **Northwestern** | | |
| **SNA** | Sella Tursica, Nasion, Point-A | The angle formed by the Sella, Nasion, and Point-A |
| **SNB** | Sella Tursica, Nasion, Point-B | The angle formed by the Sella, Nasion, and Point-B |
| **ANB** | Point-A, Nasion, Point-B | The difference between the SNA and SNB angles |
| **NAP** | Nasion, Point A, Pogonion | The angle formed by the Nasion, Point A, and Pogonion |
| **S-N-GoGn** | Sella Tursica, Nasion, Gonion, Gnathion | The angle formed by the Sella, Nasion, and Gonion-Gnathion line |
| **FH-NP** | Frankfort Horizontal to Nasion, Pogonion | The angle formed by FHP and Nasion-Pogonion line |
| **S-Gn-FH** | Sella Tursica, Gnathion, Frankfort Horizontal | The angle formed by the Sella-Gnathion line and FHP |
| **S-N-Gn** | Sella Tursica, Nasion, Gnathion | The angle formed by Sella-Nasion line and Gnathion |
| **21-NS** | Maxillary left central incisor to Nasion, Sella Tursica | The angle formed by the maxillary left central incisor and Nasion-Sella line |
| **21-31** | Maxillary left central incisor to Mandibular left central incisor | The angle formed by the upper and lower central incisors |
| **31-Go Gn** | Mandibular left central incisor to Gonion, Gnathion | The angle formed by mandibular left central incisor and Gonion-Gnathion line |
| **31 Occlusal pl.** | Mandibular left central incisor to Occlusal Plane | The angle formed by mandibular left central incisor to occlusal plane |
| **AB-Occlusal pl.** | Point-A, Point-B, to Occlusal Plane | The angle formed by Point A-Point B line, and occlusal plane |
| **21-NP** | Maxillary left central incisor to Nasion, Pogonion | The distance (mm) from the incisal edge to Nasion-Pogonion line |

| Supplementary Table 3. Multicollinearity of independent variables showed by tolerance and VIF values | | |
| --- | --- | --- |
| **Variables** | **Tolerance** | **VIF** |
| **Age** | 0.73 | 1.38 |
| **Sex** | 0.44 | 2.27 |
| **BMI** | 0.59 | 1.70 |
| **Cephalic Index** | 0.64 | 1.57 |
| **Skeletal Class** | 0.05 | 19.01 |
| **Tweed_FMA** | 0.04 | 26.27 |
| **Tweed_FMIA** | 0.02 | 46.10 |
| **Tweed_IMPA** | 0.04 | 25.56 |
| **NW_NAP** | 0.06 | 17.66 |
| **NW_SNA** | 0.06 | 15.57 |
| **NW_SN-GoGn** | 0.05 | 19.22 |
| **NW_FH-NP** | 0.11 | 9.51 |
| **NW_S-Gn-FH** | 0.17 | 5.88 |
| **NW_S-N-Gn** | 0.06 | 16.57 |
| **NW_21-NS** | 0.15 | 6.67 |
| **NW_21-31** | 0.19 | 5.34 |
| **NW_31-GoGn** | 0.07 | 14.92 |
| **NW_31-occpl** | 0.14 | 7.02 |
| **NW_AB-occpl** | 0.29 | 3.46 |
| **NW_21-NP (mm)** | 0.17 | 5.90 |

| Supplementary Table 4. Rotated Component Matrix | | |
| --- | --- | --- |
|  | **Component 1** | **Component 2** |
| **Skeletal Class** | -0.04 | 0.89 |
| **Tweed_FMIA** | 0.72 | -0.55 |
| **Tweed_IMPA** | -0.84 | 0.14 |
| **NW_21-31** | 0.80 | -0.20 |
| **NW_31-GoGn** | -0.88 | 0.15 |
| **NW_31-occpl** | 0.86 | -0.25 |
| **NW_21-NP (mm)** | -0.38 | 0.77 |
| Extraction Method: Principal Component Analysis. | | |
| Rotation Method: Varimax with Kaiser Normalization. | | |
| a. Rotation converged in 3 iterations. | |  |

| Supplementary Table 5. Regression analysis of baseline variables (age, sex, and BMI) | | | | | | | | | | | |  |
| --- | --- | --- | --- | --- | --- | --- | --- | --- | --- | --- | --- | --- |
| **No** | **Landmark** | **B0** | **B1** | **P** | **B2** | **P** | **B3** | **P** | **RMSE** | **MAE** | **R Squared** | **n** |
| 1 | Sg | 1.432 | -0.001 |  | -0.355 |  | 0.184 | ** | 1.11 | 0.83 | 0.42 | 103 |
| 2 | G | 1.665 | \| -0.002  \| \| --- \| |  | -0.284 |  | 0.163 | ** | 1.02 | 0.77 | 0.39 | 103 |
| 3 | N | 4.638 | -0.002 |  | -1.284 | ** | 0.153 | ** | 1.52 | 1.12 | 0.33 | 103 |
| 4 | Rhi | 1.137 | 0.013 | * | -0.378 | * | 0.057 | ** | 0.86 | 0.64 | 0.22 | 103 |
| 5 | Point-A | 12.404 | -0.048 | ** | -1.596 | ** | 0.106 | ** | 1.80 | 1.46 | 0.30 | 101 |
| 6 | Pr | 10.155 | -0.046 | ** | -1.267 | ** | 0.193 | ** | 1.61 | 1.30 | 0.41 | 90 |
| 7 | Id | 11.534 | -0.014 |  | -1.380 | ** | 0.140 | ** | 2.16 | 1.79 | 0.18 | 97 |
| 8 | Point-B | 9.505 | 0.003 |  | -0.617 |  | 0.140 | ** | 1.76 | 1.38 | 0.18 | 101 |
| 9 | Pog | 4.999 | -0.003 |  | -0.380 |  | 0.303 | ** | 2.58 | 1.95 | 0.25 | 103 |
| 10 | Gn | 1.729 | -0.001 |  | -0.963 |  | 0.327 | ** | 2.96 | 2.20 | 0.26 | 103 |
| 11 | Fe-L | -0.952 | 0.003 |  | -0.111 |  | 0.257 | ** | 1.41 | 1.04 | 0.44 | 103 |
| 12 | Fe-R | -0.898 | -0.008 |  | 0.083 |  | 0.265 | ** | 1.33 | 0.99 | 0.47 | 103 |
| 13 | Ft-L | -0.084 | -0.002 |  | 0.140 |  | 0.273 | ** | 1.57 | 1.16 | 0.40 | 103 |
| 14 | Ft-R | -0.465 | -0.009 |  | 0.287 |  | 0.298 | ** | 1.67 | 1.25 | 0.41 | 103 |
| 15 | Spo-L | 3.373 | -0.001 |  | -0.610 |  | 0.226 | ** | 1.62 | 1.35 | 0.35 | 103 |
| 16 | Spo-R | 3.720 | -0.011 |  | -0.523 |  | 0.235 | ** | 1.50 | 1.24 | 0.39 | 103 |
| 17 | So-L | -2.118 | 0.009 |  | 0.709 |  | 0.369 | ** | 1.86 | 1.48 | 0.47 | 103 |
| 18 | So-R | -1.105 | 0.005 |  | 0.731 |  | 0.334 | ** | 2.04 | 1.59 | 0.37 | 103 |
| 19 | Zm-L | 4.016 | -0.039 | * | 0.561 |  | 0.568 | ** | 2.64 | 2.04 | 0.51 | 103 |
| 20 | Zm-R | 4.547 | -0.039 | * | 0.218 |  | 0.559 | ** | 3.05 | 2.27 | 0.44 | 103 |
| 21 | Zy-L | -6.147 | -0.026 |  | 1.657 | ** | 0.600 | ** | 2.70 | 1.91 | 0.52 | 102 |
| 22 | Zy-R | -5.915 | -0.032 |  | 2.468 | ** | 0.595 | ** | 2.78 | 2.08 | 0.52 | 103 |
| 23 | Kdl-L | -2.509 | -0.038 |  | -0.011 |  | 0.888 | ** | 3.82 | 2.91 | 0.55 | 102 |
| 24 | Kdl-R | -1.470 | -0.056 | * | 0.675 |  | 0.873 | ** | 4.05 | 3.08 | 0.51 | 103 |
| 25 | Mm-L | -0.447 | -0.010 |  | 0.694 |  | 1.022 | ** | 3.86 | 2.95 | 0.61 | 102 |
| 26 | Mm-R | 5.091 | -0.048 | * | 0.266 |  | 0.902 | ** | 4.15 | 3.29 | 0.52 | 103 |
| 27 | Go-L | -15.081 | -0.006 |  | 2.372 |  | 1.275 | ** | 5.50 | 4.30 | 0.50 | 101 |
| 28 | Go-R | -7.481 | -0.022 |  | 0.674 |  | 1.130 | ** | 5.76 | 4.65 | 0.43 | 102 |
| 29 | Spm2-L | 18.544 | -0.017 |  | -1.542 |  | 0.783 | ** | 5.87 | 4.56 | 0.31 | 72 |
| 30 | Spm2-R | 10.850 | -0.034 |  | 1.764 |  | 0.957 | ** | 6.28 | 5.03 | 0.27 | 71 |
| 31 | Sm2-L | 13.446 | -0.004 |  | -0.357 |  | 0.854 | ** | 5.18 | 4.19 | 0.36 | 66 |
| 32 | Sm2-R | 13.905 | 0.030 |  | 0.621 |  | 0.757 | ** | 5.34 | 4.15 | 0.30 | 66 |
| 33 | Apc-L | 10.068 | -0.024 |  | -1.498 | ** | 0.235 | ** | 2.11 | 1.73 | 0.33 | 100 |
| 34 | Apc-R | 11.240 | -0.026 |  | -2.147 | ** | 0.221 | ** | 2.19 | 1.67 | 0.35 | 98 |
| 35 | Al-L | 9.430 | -0.022 |  | -0.932 |  | 0.311 | ** | 3.34 | 2.87 | 0.19 | 102 |
| 36 | Al-R | 9.955 | -0.020 |  | -1.474 | * | 0.297 | ** | 3.20 | 2.66 | 0.22 | 102 |
