# Supplementary material for: Assessing the contribution of orthodontic profiles in predicting facial soft tissue thickness for forensic facial approximation
Source: Int J Legal Med. 2025 Jun 18;139(6):2951–64. doi: 10.1007/s00414-025-03542-x (PMC12532767; doi:10.1007/s00414-025-03542-x)
Supplement: Supplementary file 2 — Supplementary Material 2 [file 414_2025_3542_MOESM2_ESM.docx]

SUPPLEMENTARY FILE 2

Supplementary Fig. 1. Histogram for Descriptive Statistics

Supplementary Fig. 2. Visualization of metrics comparison between baseline models and PCA models. A) RMSE

Supplementary Fig. 2. Visualization of metrics comparison between baseline models and PCA models. B) MAE

Supplementary Fig. 2. Visualization of metrics comparison between baseline models and PCA models. C) R squared

Supplementary Fig. 3. Visualization of metrics comparison between BMI-based mean estimates and PCA models. A) RMSE

Supplementary Fig. 3. Visualization of metrics comparison between BMI-based mean estimates and PCA models. B) MAE
